# Hypoxia alters the century old Berger effect

**DOI:** 10.3389/fnins.2026.1754436

**Published:** 2026-01-26

**Authors:** Evan Hutcheon

**Affiliations:** ImageTech, Simon Fraser University, Surrey, BC, Canada

**Keywords:** alpha band power, alpha power, Berger effect, EEG, hypoxia, hypoxia and EEG

## Introduction

The alpha rhythm was the first discovered canonical frequency band, first identified by Hans Berger almost a century ago (Berger, 1929). Berger observed that alpha activity increased during eyes-closed resting state and decreased during eyes-open resting state, with this phenomenon known as the Berger effect. Despite nearly a century of investigation, the literature is divided on the neurophysiological origins and functional significance of the alpha band. Alpha oscillations have been linked to cognition (Khader and Rösler, 2011; Doesburg et al., 2016), the regulation of cortical excitability (Sauseng et al., 2009a), hypoxia (Hutcheon et al., 2023), and various disorders (Eidelman-Rothman et al., 2016; Carter Leno et al., 2018).

A growing body of evidence indicates that alpha activity is highly sensitive to hypoxia (Gritti et al., 2012; Kraaier et al., 1988; Ozaki et al., 1995; Rice et al., 2019; Schellart and Reits, 2001; Schneider and Strüder, 2009; Zhao et al., 2016). Several studies report an almost reversal of the Berger effect under hypoxic conditions (Kraaier et al., 1988; Ozaki et al., 1995; Schellart and Reits, 2001), providing a unique model for probing the mechanisms underlying alpha generation. In this Opinion piece, I discuss how hypoxia alters alpha activity, highlight inconsistencies in the literature, and argue that examining the alpha frequency band under hypoxia may offer critical insights into the neurophysiology of alpha oscillations.

## Hypoxia, alpha and the Berger effect

Multiple studies have found that hypoxia reduced alpha power during eyes-closed resting state (Gritti et al., 2012; Kraaier et al., 1988; Ozaki et al., 1995; Schellart and Reits, 2001) and increased alpha during eyes-open resting state (Schellart and Reits, 2001; Zhao et al., 2016), contradicting the classic Berger effect. This reversal; however, is not consistently observed (Hutcheon et al., 2023; Schneider and Strüder, 2009; Coronel-Oliveros et al., 2024). Differences in altitude, duration of exposure, acclimatization, type of hypoxic exposure, and experimental design all contribute to the contradictory findings on the hypoxic impact on alpha power.

Discrepancy in alpha power changes due to hypoxia have been found during both resting state, and experimental tasks. A study utilizing source-localized electroencephalogram (EEG) hypothesized to find changes in alpha power but instead reported only significantly increased beta-1 power (Schneider and Strüder, 2009), contrasting with earlier eyes-closed resting state sensor-space studies (Kraaier et al., 1988; Ozaki et al., 1995). The differing findings in this study may be due to a lower level of hypoxia (13,000 ft compared to 25,000 ft) than the aforementioned studies, alongside their source-space analysis. Alongside resting-state studies, discrepancies in results have been found with similar experimental paradigms. A study utilizing a flight-simulation task found normobaric hypoxia to reduce power across all frequency bands at an equivalent level of hypoxia at 25,000 ft (Rice et al., 2019), whereas a previously performed flight-simulation task found hypoxia to increase power across all frequency bands during hypobaric hypoxia equivalent to 25,000 ft. These contrasting results may be due to differences in EEGs, one used a dry-EEG setup with only seven electrodes (Rice et al., 2019) and the other 19 electrodes (Papadelis et al., 2007), alongside one study utilizing normobaric hypoxia and the other utilizing hypobaric hypoxia. These examples highlight the difficulty in understanding the impact of hypoxia on alpha power; a discrepancy in the direction and magnitude of alpha power change is found both during task and resting state studies.

## Hypoxia as a probe of alpha's neurophysiological origins

The classical interpretation of eyes-closed alpha as a “default” visual rhythm generated in the absence of visual input has been challenged by work showing that alpha can be modulated during eyes-closed states. It has been demonstrated that motion-sensitive visual processes continue to influence alpha generation during eyes-closed states (Hohaia et al., 2022), indicating that alpha is not a passive idling rhythm but an active oscillatory process subject to ongoing modulation. This is consistent with hypoxia studies showing modulated (i.e., decreased in these cases) eyes-closed alpha power (Ozaki et al., 1995; Schellart and Reits, 2001) and adds evidence to the notion that eyes-closed alpha power might not be a pure “default” setting.

Hypoxia has been shown to redistribute alpha topography. During a visuospatial attention task, it was reported that alpha activity shifted from a primarily occipital distribution under normoxia to a right-lateralized occipitoparietal pattern under hypoxia, interpreted as compensatory recruitment to sustain attention (Zani et al., 2020). Such findings suggest that hypoxia does not merely alter alpha power but could modify the spatial configuration of alpha generators.

Alpha oscillations have been consistently linked to core cognitive functions, including sustained attention (Lenartowicz et al., 2018), visuospatial orienting (Doesburg et al., 2016, 2009), memory processes (Pavlov and Kotchoubey, 2022; Sauseng et al., 2009b), alongside disorders such as attention-deficit/hyperactive disorder (ADHD) (Deiber et al., 2020) and Alzheimer's disease (Wang et al., 2015). Beyond alpha powers functional associations, alpha rhythms are thought to contribute to the homeostatic regulation of cortical excitation and inhibition, with both increases and decreases in alpha power reflecting shifts in inhibitory gating and cortical gain control (Haegens et al., 2011; Klimesch, 2012; Mathewson et al., 2011). Hypoxia produces well-documented impairments in attention and memory (Wang et al., 2022), as well as disruptions in visuospatial orienting (Zani et al., 2020). As hypoxia reduces oxygen availability and consequently ATP production, it may disturb excitatory–inhibitory balance in cortical networks. Given alpha's central role in these cognitive operations, hypoxia-induced cognitive deficits are likely mediated, at least in part, through alterations in alpha dynamics.

## Mechanistic considerations: glucose metabolism and cholinergic pathways

Hypoxia studies also provide evidence of the metabolic contributions to alpha activity. After 63 days at altitude, decreased glucose metabolism was observed in frontal regions, the left occipital lobe, the right thalamus, and increased metabolism in the cerebellum (Hochachka et al., 1999). A theory for alpha generation is that thalamic pace maker cells located in the lateral geniculate nucleus, are triggered by cholinergic modulation resulting in occipital alpha as seen with an EEG (Sharma and Nadkarni, 2020). Occipital alpha is highly synchronized with thalamic local field potentials (Hughes, 2011), and cholinergic modulation plays a key role as cholinergic antagonists applied to the lateral geniculate nucleus reduced occipital alpha power in cats (Lorincz et al., 2009). Hypobaric hypoxia alters muscarinic acetylcholine receptors in rodent prefrontal cortex following 6–24 h of exposure (Sharma et al., 2025), muscarinic antagonists such as scopolamine have also been found to impact alpha power in humans (Osipova et al., 2003), with a reduction in eyes-open/eyes-closed ratio found prominently in posterior regions implying a scopolamine induced lack of desynchronization during the eyes-open state. This is similar to the lack of eyes-open desynchronization found by some groups during hypoxia (Schellart and Reits, 2001; Zhao et al., 2016). This raises the possibility that acetylcholine receptor downregulation induced by hypoxia (Sharma et al., 2025) may contribute to the abnormal alpha pattern found during hypoxia. It would be of interest to compare scopolamine and hypoxia, and whether both produce similar abnormal alpha rhythms. Hypoxia-induced downregulation of glucose metabolism, combined with acetylcholine receptor down regulation, may contribute to the atypical alpha patterns observed in hypoxia.

## Arousal's impact on hypoxic alpha

An individual's state of arousal is another critical but often under appreciated factor in interpreting hypoxia-related changes in alpha power. Alpha power is tightly linked to fluctuations in arousal and sleep disruption, both of which are altered during sustained hypoxia exposure. Thus, apparent “reversals” of the Berger effect may partially reflect hypoxia-induced changes in arousal rather than hypoxia. Sleep disruption is particularly relevant in field studies at altitude, where hypoxia is accompanied by fragmented sleep, periodic breathing, and changes in sleep (Fabries et al., 2022). Although acute laboratory hypoxia does not involve participants sleeping while hypoxic, individual differences in arousal state can interact with alpha dynamics. One study comparing hypoxia with sleep deprivation found that lower alpha (7–9 Hz) and not higher alpha (10–12 Hz), measured during pre-stimulus, decreased during hypoxia alone, but not when hypoxia was combined with tiredness, suggesting that the well-documented increase in alpha during fatigue may counteract hypoxia-related alpha reductions (Jacques et al., 2025). Interestingly, they found no alteration to event-related alpha potentials during an attention task, contradicting the findings of a previous study during a visuospatial attention task that found an increase in event-related alpha (Zani et al., 2020). This contradiction in findings highlights the complexity of comparing hypoxia studies. The difference in results may be due to different levels of hypoxia (3,500 m compared to 4,200 m), suggesting that one study may have not had a severe enough level of hypoxia to impact alpha power. Sleep and arousal may act as confounding variables that could potentially mask or potentiate the direct effects of hypoxia. Systematic control and measurement of arousal and sleep quality are therefore essential for interpreting alpha changes in hypoxia research.

## Hypoxic alpha has similarities with aging

Understanding the impact of hypoxia on alpha can potentially lead to a better understanding of certain aspects of aging. Eyes-open hypoxia caused increased relative theta, decreased relative alpha, and flattening of the 1/f slope during, particularly in occipitoparietal and front central regions (Coronel-Oliveros et al., 2024). This study was performed during acute hypoxia, and in sensor space, with the decrease in alpha contradicting previous studies that found an increase in alpha during eyes-open resting state (Schellart and Reits, 2001; Zhao et al., 2016). Their results mirrored some of the features of aging (Coronel-Oliveros et al., 2024): reduced alpha power (Tröndle et al., 2023), and 1/f slope flattening have been observed in dementia (Neto et al., 2016) and healthy aging (Tröndle et al., 2023; Park et al., 2024; Scally et al., 2018). These similarities between hypoxia and aging suggest that hypoxia may offer insights into certain aspects of aging in terms of alpha power and 1/f slope; however, we first must understand the nuances involved in alpha power during hypoxia.

## Discussion

Alpha oscillations play a central role in cognitive function and are disrupted across multiple disorders, including Alzheimer's disease and ADHD. Hypoxia offers an experimentally controlled way to disrupt the alpha rhythm and probe its underlying mechanisms. Notably, hypoxia may even reverse the century-old Berger effect, challenging foundational assumptions about how alpha is generated and modulated.

At present, inconsistencies across the literature remain substantial. Differences in severity and duration of hypoxia, type of hypoxia, arousal state, sleep quality, task context, and methodological differences (e.g., sensor or source space; resting state or task based; dry electrodes or wet electrodes) all complicate interpretation. The possibility that alpha reversal occurs only at severe hypoxia warrants systematic testing.

A clearer understanding of why hypoxia alters the classic Berger effect may reveal fundamental principles of alpha generation, illuminate parallels with aging and Alzheimer's disease, and establish hypoxia as a powerful model for studying the neurophysiology of human cortical oscillations.

The first step would be to replicate previous work (Schellart and Reits, 2001) by completing a direct comparison of eyes-open and eyes-closed during hypoxia, then to discern what is the hypoxic cut-off for this effect. The Berger effect has been a foundation of EEG research for almost a century, so why does hypoxia potentially disturb this foundational effect of EEG research?
